# Comparison of Postoperative Pain Management Between Saphenous Nerve Block and High-Volume Proximal Adductor Canal Block for Arthroscopic Knee Surgery

**DOI:** 10.5812/aapm-163154

**Published:** 2025-12-31

**Authors:** Ali Khatibi, Mahmood-Reza Alebouyeh, Maryam Aavin Salahian, Ali Torkaman, Poupak Rahimzadeh, Seyed Hamid Reza Faiz

**Affiliations:** 1 Department of Anesthesiology, School of Medicine, Iran University of Medical Sciences, Tehran, Iran; 2 Rasoul-Akram Medical Center, Iran University of Medical Sciences, Tehran, Iran; 3 Iran University of Medical Sciences, Tehran, Iran; 4 Department of Orthopedics, Iran University of Medical Sciences, Tehran, Iran; 5 Department of Anesthesiology and Pain Medicine, Rasoul-Akram Medical Center, Iran University of Medical Sciences, Tehran, Iran; 6 Minimally Invasive Surgery Research Center, School of Medicine, Iran University of Medicine Sciences, Tehran, Iran

**Keywords:** Lower Limb Nerve Blocks, Saphenous Nerve Block, Knee Surgery, Postoperative Pain Management, Post Operation Acute Pain, Pain Management

## Abstract

**Background:**

Lower limb surgeries are typically accompanied by severe postoperative pain, and managing such pain is of great importance. Inadequate pain management can lead to serious complications such as myocardial ischemia and impaired pulmonary function. To manage pain, local anesthetic techniques have been introduced as effective methods. One of these techniques is the high-volume proximal adductor canal block (HI-PAC), in the distal third of the medial thigh, which directly targets the femoral nerve and indirectly the sciatic nerve.

**Methods:**

In this double-blind study, patients aged 30 to 70 years with American Society of Anesthesiologists (ASA) physical status I and II who underwent anterior cruciate ligament (ACL) reconstruction were divided into two groups. Both groups received general anaesthesia using the same method. The control group received an ultrasound-guided saphenous nerve block in the proximal third of the anteromedial thigh with 0.2% ropivacaine (15 mL), while the case group received a HI-PAC in the distal third of the thigh with 0.1% ropivacaine (30 mL). Pain intensity and analgesic effectiveness were evaluated at predetermined time points (baseline, 0.5, 2, 4, 6, and 12 hours post-block). Pain severity was assessed using the Numeric Rating Scale (NRS), and agitation was measured with the Ramsay Sedation Score. Data were analyzed using appropriate statistical tests via SPSS version 26.

**Results:**

A total of 50 patients participated: 24 in the case group (HI-PAC block) and 26 in the control group (saphenous nerve block). In terms of pain scores (NRS), the initial pain score was 9.20 in group A and 9 in group S. Pain intensity decreased significantly over time in both groups. At 0.5, 2, 4, 6, and 12 hours after the block, the average NRS score was 4.01 in group A and 4.18 in group S.

**Conclusions:**

The mean opioid consumption and level of agitation were similar in both groups. Multivariate analysis indicated that both nerve block techniques were equally effective in reducing acute postoperative pain, and the type of block did not have a statistically significant effect on pain severity.

## 1. Background

Lower limb surgeries are among the surgical procedures that are typically accompanied by severe postoperative pain. Therefore, managing this pain and identifying the factors that influence its reduction is of great importance to ensure the most effective pain control with the least complications and the most appropriate analgesic technique for patients (1). Studies have shown that more than 50% of patients undergoing arthroplasty complain of considerable postoperative pain (2). If postoperative pain is not controlled, it may result in numerous complications such as myocardial ischemia, impaired lung function, paralytic ileus, urinary retention, thromboembolic events, infection due to immune dysfunction, anxiety, and ultimately chronic pain that can persist for more than three months after surgery (3). Furthermore, inadequate pain management can lead to patient dissatisfaction, hinder rehabilitation, and prolong hospitalization (3). Poorly uncontrolled acute postoperative pain is an important predictive factor in the development of chronic persistent post-surgical pain (CPSP) (4). Pain management in such surgeries remains a challenging topic, and several methods have been introduced for this purpose (5). Among them, the ideal method is the one that ensures optimal pain control and the widest possible nerve coverage involved in pain generation.

The effectiveness of local anesthetic techniques as part of a multimodal approach to improving postoperative pain control has been well established. Achieving proper pain control through effective nerve blocks is especially important in knee surgeries (6, 7).

The ideal technique is one that has minimal complications and produces rapid results, thereby reducing the use of opioids and other analgesics with significant side effects.

The High-Volume Proximal Adductor Canal Block (HI-PAC) is a novel technique introduced for managing pain following knee surgeries. This method involves a single injection that directly targets the saphenous nerve within the proximal adductor canal and indirectly affects the sciatic nerve.

This block is also described as an important sensory technique in which a large volume of local anesthetic is used to reach the popliteal space indirectly through an anterior approach to the sciatic nerve. Proponents of this technique claim that increasing the volume of the anesthetic leads to its spread toward the popliteal fossa, thereby affecting the branches of the sciatic nerve in that region.

## 2. Methods

This study was a randomized, double-blind clinical trial conducted to compare the effectiveness of two techniques — saphenous nerve block with the standard drug dose and proximal adductor canal block with high-volume drug (HI-PAC) — for controlling acute pain in patients undergoing anterior cruciate ligament (ACL) reconstruction at Rasoul-Akram and Firoozgar hospitals.

Inclusion Criteria: Patients aged 30 to 70 years, American Society of Anesthesiologists (ASA) class I or II, undergoing ACL reconstruction surgery, without a history of opioid addiction, without having taken any analgesics in the last 48 hours, and no prior surgeries on the operated knee.

Exclusion Criteria: Surgery duration exceeding three hours; altered consciousness postoperatively that would interfere with patient response; absence of severe pain in the recovery room that would make nerve block unnecessary.

Patients were randomly assigned before anesthesia, using a random number table, into one of the two groups: saphenous nerve block or HI-PAC.

Anesthesia Method: All patients received general anesthesia consisting of premedication with fentanyl (2 mcg/kg), midazolam (2 mg), and induction with propofol (2 mg/kg) and cisatracurium (0.2 mg/kg). Maintenance included a continuous infusion of propofol (50 - 150 mcg/kg/min), with repeated doses of fentanyl (1 mL every hour) and cisatracurium (2 mg every 30 minutes) until the end of surgery.

### 2.1. Postoperative Procedure and Group Interventions

After completion of surgery and transfer of the patient to the recovery room, if the patient experienced pain and provided informed consent, their initial pain score was recorded.

The control group (Group S) underwent a saphenous nerve block as follows: the patient was placed in the supine position, and the leg to be blocked was slightly externally rotated to expose the upper thigh. Under fully sterile conditions, a high-frequency linear ultrasound probe (15 - 6 MHz) was placed transversely over the proximal third of the anteromedial thigh to obtain a short-axis view of the cross-section. The femoral artery was identified as a sonographic landmark on the medial side, and using a 22-gauge needle in an in-plane approach relative to the probe, the needle was advanced under ultrasound guidance to a depth of approximately 2 to 3 cm toward the saphenous nerve. After confirming the correct position, 15 mL of 0.2% ropivacaine was injected lateral to the femoral artery beneath the sartorius muscle.

The case group underwent the HI-PAC as follows: the patient was positioned supine with greater external rotation of the leg. The ultrasound probe was placed transversely over the distal third of the medial thigh under sterile conditions to obtain a short-axis view of the canal. Using a 22-gauge needle in an in-plane approach relative to the ultrasound probe, the needle was guided to a depth of approximately 2 to 3 cm under sonographic visualization. After confirming proper positioning, 30 mL of 0.1% ropivacaine was injected into the subsartorial vascular sheath (adductor canal fascia).

### 2.2. Post-block Assessments

Pain intensity was evaluated at 0.5, 2, 4, 6, and 12 hours after the block using the Numeric Rating Scale (NRS). Agitation was measured at 2, 4, 6, and 12 hours post-block using the Ramsay Sedation Score. Opioid consumption was documented based on administration of 5 mg of morphine when moderate-to-severe pain (pain score higher than 4) was present, as per physician orders. The total opioid dose used was recorded at various time points until discharge.

### 2.3. Data Analysis

Data from this study were analyzed using SPSS version 26. Central and dispersion indices were described depending on the variable type, using mean, median, mode, standard deviation, and interquartile range (IQR). Data were presented in tables and figures. Continuous variables were reported as mean ± standard deviation with 95% confidence intervals, and categorical variables were expressed as frequency and percentage.

## 3. Results

**Figure 1.**
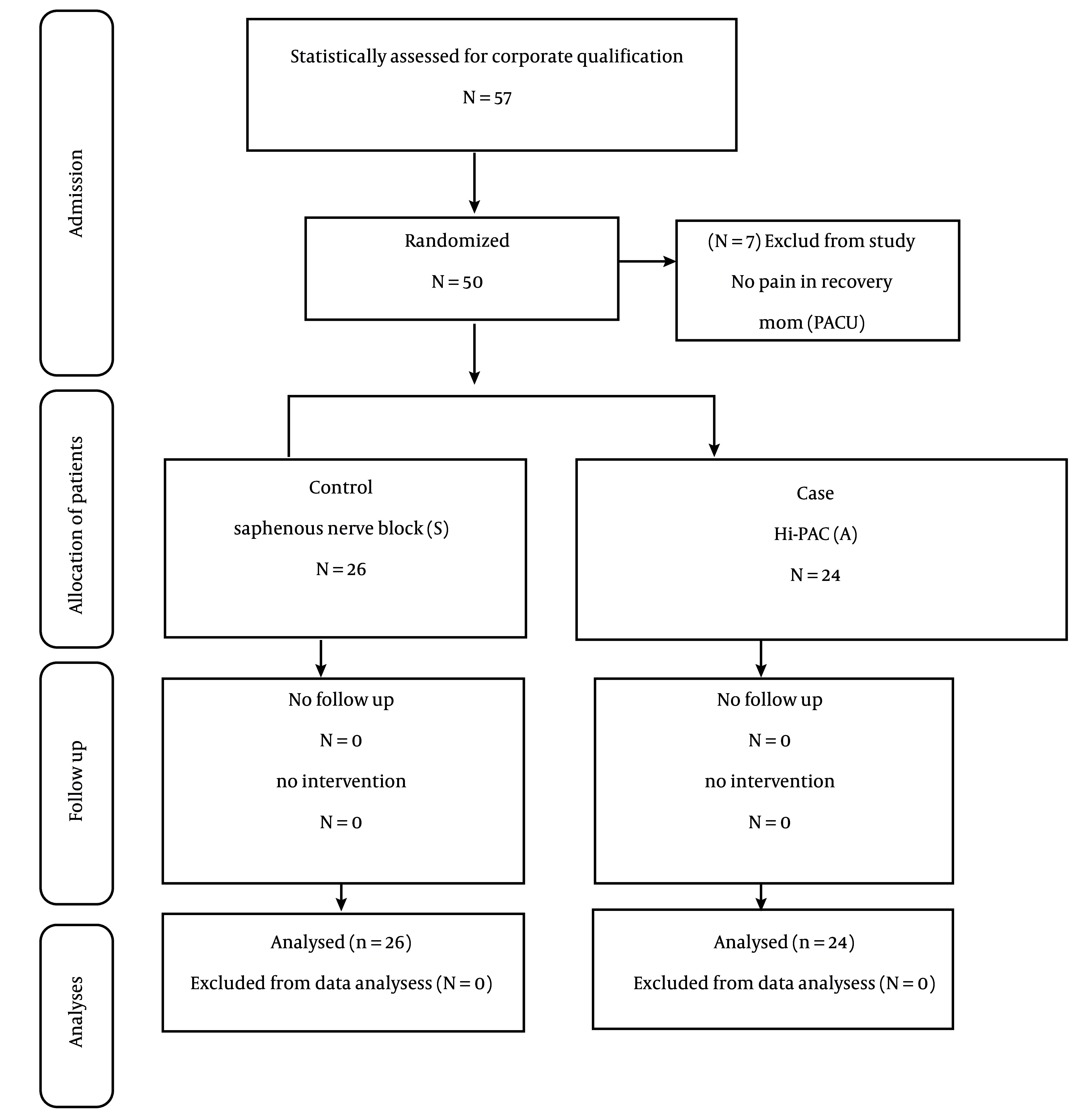
Consort flow chart of present study

In this study, 57 patients who met the inclusion criteria and gave informed consent were enrolled. Seven patients were excluded due to lack of pain in the recovery room, leaving 50 participants (Figure 1). 

In the HI-PAC group (Group A), there were 24 patients, while in the saphenous block group (Group S), there were 26 patients. Gender distribution: 28 females (56%), 22 males (44%). Age distribution: comparable between the two groups. The ASA classification: similar between groups (Table 1). 

**Table 1.** Demographic Information ^a^

Variables	A	S	P-Value
**Gender**			0.80
Men	11 (45.8)	11 (42.3)	
Women	13 (54.1)	15 (57.6)	
**Age (y)**	46.42 ± 12.54	45.96 ± 11.51	00.89
**Duration of operation (min)**	131.25 ± 30.40	132.69 ± 27.06	00.86
**ASA**			0.5
1	23 (95.8)	21 (80.7)	
2	1 (4.16)	5 (19.2)	

Abbreviation: ASA, American Society of Anesthesiologists.

^a^ Values are expressed as No. (%) or mean ± SD.

**Figure 2.**
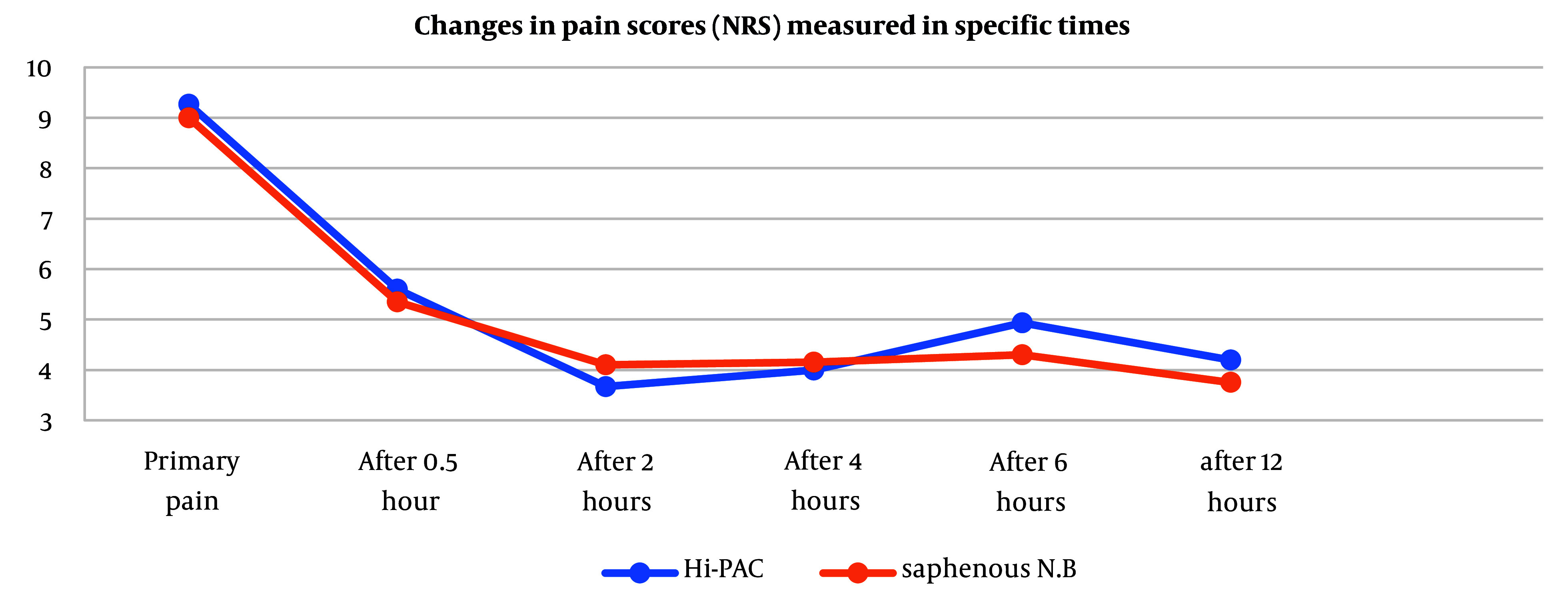
Changes in pain score (NRS) measured at specific times (Abbreviation: NRS, Numeric Rating Scale)

The overall average pain scores during the measured time intervals were similar: Saphenous group: 4.18, HI-PAC group: 4.01 (P-value = 0.520, indicating no significant difference). The initial pain scores were: Group S: 9.0, Group A: 9.20. Furthermore, the mean pain scores measured at 0.5, 2, 4, 6, and 12 hours postoperatively indicated a decreasing trend in both block groups. In the detailed comparison of pain severity between the two groups at each of these time points, no statistically significant differences were observed. The P-values at 0.5, 2, 4, 6, and 12 hours post-block were respectively 0.683, 0.373, 0.134, 0.382, and 0.380 (Figure 2). 

According to descriptive statistics and results from multivariate analysis, the average opioid consumption at various monitored time points was similar between the two nerve block techniques (HI-PAC and saphenous nerve block). Neither of the two blocks demonstrated a statistically significant advantage in terms of reduced opioid use during the first 12 hours after surgery. The mean opioid consumption in the saphenous block group was 3.54 mg morphine and in the HI-PAC group was 3.14 mg morphine with P-value = 0.4 (Table 2). 

**Table 2.** Mean Opioids Consumption ^a^

Mean Opioids Consumption	No.	Mean ± SD	P-Value
**Block type**		3.54 ± 2.84	0.422
A	24		
S	26	4.23 ± 3.14	

^a^ Morphine in mg.

Additionally, when evaluating agitation at 2, 4, 6, and 12 hours post-block, the obtained P-values were 0.86, 0.32, 0.40, and 0.57, respectively—indicating no significant difference in agitation control between the two block methods (Tables 3 to 6).

**Table 3.** Agitation Score After 2 Hours ^a^

Ramsy Score After 2 h	Blocks		P-Value
	A	S	
**1**	7 (29.2)	7 (26.9)	0.86
**2**	17 (70.8)	19 (73.0)	
**Total**	24	26	

^a^ Values are presented as No. (%).

**Table 4.** Agitation Score After 4 Hours ^a^

Ramsy Score After 4 h	Blocks		P-Value
	A	S	
**1**	5 (20.8)	13 (50.0)	0.32
**2**	19 (79.1)	13 (50.0)	
**Total**	24	26	

^a^ Values are presented as No. (%).

**Table 5.** Agitation Score After 6 Hours ^a^

Ramsy Score After 6 h	Blocks		P-Value
	A	S	
**1**	13 (54.2)	11 (42.3)	0.40
**2**	11 (43.8)	15 (57.7)	
**Total**	24	26	

^a^ Values are presented as No. (%).

**Table 6.** Agitation Score After 12 Hours ^a^

Ramsy Score After 12 h	Blocks		P-Value
	A	S	
**1**	13 (54.1)	12 (48.0)	0.57
**2**	11 (45.8)	14 (53.8)	
**Total**	24	26	

^a^ Values are presented as No. (%).

## 4. Discussion

The results of this study indicated that both techniques under investigation were effective in reducing pain intensity over time; however, there was no statistically significant difference between the two groups in terms of changes in pain severity. Similarly, agitation levels and opioid consumption were also found to be comparable between the groups.

These findings are in line with the study by Sonawane et al., who, using two different volumes of anesthetic in the adductor canal, demonstrated that both methods produced effective analgesia, with most patients remaining comfortable and pain-free until discharge (3). Likewise, the study by Sveom et al., which compared saphenous nerve blocks in the upper and lower parts of the adductor canal in patients undergoing total knee replacement under ultrasound guidance, found that both techniques were equally effective in controlling pain and reducing opioid use, with no significant difference between the two groups (8). These results are consistent with the present study.

In a similar vein, Kulkarni et al., in a single-blind randomized clinical trial, reported higher Visual Analog Scale (VAS) scores in patients who received adductor canal blocks compared to intraoperative infiltration (9). In contrast, a study by Rahimzadeh et al. showed that conventional femoral nerve block at the inguinal canal resulted in lower analgesic requirements, higher patient satisfaction, and greater pain relief compared to the adductor canal block (2).

What sets the present study apart from previous research is its specific focus on the HI-PAC, using a local anesthetic dose equal to that of the saphenous group, but with half the concentration and double the volume — aiming for drug spread into the popliteal region to potentially block branches of the sciatic nerve. This hypothesis was not supported by the results. It appears that the low concentration or insufficient volume used in the HI-PAC technique may have contributed to the lack of superior outcomes and, on the other hand, the spread of anesthetic to the sciatic nerve territory may not have occurred despite the larger volume.

Overall, the results of this study demonstrate that both the saphenous nerve block and the HI-PAC are safe and effective methods for controlling acute pain following knee surgery. However, there was no significant difference between the two in terms of reducing pain severity or opioid consumption. Considering the findings of this study, the saphenous block — performed at the proximal third of the thigh with a lower volume of anesthetic and without the need to remove the knee splint or apply significant external rotation — appears to be technically easier and more comfortable for both the physician and the patient. In contrast, the HI-PAC block requires greater external rotation of the painful knee, removal of the splint, and use of a higher drug volume, making it relatively more demanding.

### 4.1. Suggestions

-Larger studies with bigger sample sizes should be conducted to examine potential differences between the two methods more precisely.

-Inclusion of a control group without any drug intervention

-Studies to investigate the effect of these two methods on chronic pain following knee surgeries

-A comparative evaluation in terms of motor block

-It is suggested to perform the adductor canal block with higher volumes and concentrations, for example, with 40 cc of 0.2% ropivacaine.

## Data Availability

The dataset presented in the study is available on request from the corresponding author during submission or after publication. The data are not publicly available due to patient privacy.

## Clinical Trial Registration Code:

IRCT20210607051501N2.
